# Plasma Jets Fabricated in Low-Temperature Cofired Ceramics for Gold Nanoparticles Synthesis

**DOI:** 10.3390/ma13143191

**Published:** 2020-07-17

**Authors:** Olga Rac-Rumijowska, Jan Macioszczyk, Tomasz Matusiak, Leszek Golonka, Helena Teterycz

**Affiliations:** Faculty of Microsystem Electronics and Photonics, Wroclaw University of Science and Technology, 50-370 Wroclaw, Poland; jan.macioszczyk@pwr.edu.pl (J.M.); tomasz.matusiak@pwr.edu.pl (T.M.); leszek.golonka@pwr.edu.pl (L.G.); helena.teterycz@pwr.edu.pl (H.T.)

**Keywords:** atmospheric pressure plasma jets, low temperature cofired ceramics, gold nanoparticles synthesis

## Abstract

In this article, we present a development of atmospheric pressure plasma jets (APPJs) for modification of liquid solutions. APPJs were fabricated in low temperature cofired ceramics (LTCC) technology. During the measurements, plasma jets worked under various flowing gases, which can be used to produce plasma activated water. In addition, owing to the plasma treatment, it was possible to decrease the time of a synthesis of gold nanoparticles (AuNPs) without the use of additional hazardous reagents. The mechanism of gold nanoparticles formation in cold nitrogen plasma is also presented.

## 1. Introduction

Plasma is one of the four fundamental states of matter which is an ionized gas exhibiting collective behavior. It can be generated by delivering sufficient amount of energy in a specific form, e.g., in electrical DC and AC discharges. Cold plasma jets are the most interesting among other plasma devices. They have numerous advantages such as generation of plasma under atmospheric pressure, simple construction and a variety of properties of the generated plasma [1]. They can be used for the treatment of solids or liquids surfaces, their modification, biologic sterilization or chemical synthesis [2]. Typical plasma jets are made by means of fine mechanics. They have some drawbacks, such as high power consumption and generation of thermal (hot) plasmas. Hot plasmas reach temperatures of more than 10,000 K, which is required in spraying of high melting point metals and alloys, but it excludes them from other applications.

In the recent 20 years, plasmas with dimensions smaller than one millimeter, have been generated in atmospheric pressures. This reduces the cost of a plasma device and makes it available for more applications. The type of an application determines the construction of jets and, as a result, properties of the generated plasma. There are many types of APPJs based on the arrangement of electrodes and frequency of discharge voltage. Obviously, not all of the materials remain unaffected by the plasma treatment, but ceramics and glass do.

Ceramic materials are resistant to chemical compounds, high voltages and high temperatures during the plasma jet operation [3]. The low temperature cofired ceramics (LTCC) technology combines these advantages with the possibility of 3D structures fabrication and easy deposition of conductive paths. It is one of the microelectronic technologies which were developed for manufacturing microfluidics systems [4]. The ability of LTCC to fabricate multilayer circuits opens this technology to microsystem technology (MST), which is applied in the production of various sensors [5]. The LTCC has already been used for fabrication of plasma generators [5,6,7,8] for s elements in liquidous solutions [6,7,8,9] or flowing gas [10].

In the literature there are numerous examples of the synthesis of plasma-assisted gold nanoparticles (AuNPs). These methods most often differ with the type of plasma generator used or the stage of synthesis at which the plasma was applied. Chloroauric acid is commonly used as a precursor of gold ions. Most often, the reduction of gold ions occurs at the plasma–liquid interface or directly in the liquid when one of the electrodes is immersed in the reaction solution. As a result of a plasma treatment, various nitrogen compounds are formed. They have good solubility in water, which significantly lowers the pH of the plasma activated water (PAW). In case of oxygen plasma, the $Au^{3+}$ ions reduction is conducted in the presence of reactive $H^{+}$, $H^{-}$, $O^{-}$, $OH^{-}$, $H^{˙}$, $O^{˙}$ and $H_{2}O_{2}$ radicals [11]. However, the mechanism of the influence of plasma on the formation of gold nanoparticles is not well understood and in many publications one can find various proposals for the reaction of the formation of gold nanoparticles. According to some sources, various types of reactive nitrogen and oxygen or high-energy electrons can act as gold-ion reducers. All of these particles or their mixtures reduce gold ions and replace traditional, often toxic, reducing agents [11]. The mechanism of obtaining gold nanoparticles in oxygen plasma has been described many times, however, there are few reports on the preparation of gold nanoparticles in nitrogen plasma. This publication will propose possible reactions of reducing gold ions in a cold nitrogen plasma environment.

A synthesis as a result of plasma action in water, ethanol or liquid nitrogen makes it possible to obtain AuNPs with a dimension of the order of several nm [12,13]. In an aqueous solution at ambient temperature using glow discharge plasma allows the formation of gold nanoparticles as a result of plasma plating of an aqueous solution of chloroauric acid without the addition of stabilizers or reducing agents. The particle size obtained in such conditions increases with the concentration of gold ions [14]. High-energy electrons can also play an important role in stabilizing nanoparticles. Liang et al. [15] postulate that, as a result of plasma plating, gold nanoparticles with a negative surface charge, which can repel each other preventing agglomeration, are formed. Wang et al. [16] obtained gold nanoparticles in water with the atmospheric-pressure nonthermal microplasma method. Sodium citrate was used as a reducer. They determined that the AuNPs size distribution is affected by the mixing mode, stabilizer concentration and plasma power. Plasma-assisted synthesis can also be carried out in non-aqueous solutions such as dodecane containing sodium bis (2-ethylhexyl) sulfosuccinate as a stabilizer [17].

Nanoparticles can be synthesized in flow reactors [18,19] or by plasma plating of a constant volume of solution, as in this publication. The use of plasma as a gold ion reducing agent in aqueous solutions without the use of additional reducing agents (such as sodium borohydride) has increasing medical applications. The nanoparticles formed with this method can be used in imaging, drug transport and cancer therapy [12]. In addition, most methods that use plasma do not require the use of expensive and advanced equipment, and the gold nanoparticles obtained in cold atmospheric plasma have a synergistic anticancer effect [12].

The main purpose of the article was to present the mechanism of gold nanoparticles synthesis in the presence of plasma. In the first stage using an atmospheric pressure plasma jets (APPJs) plasma activated water (PAW) was obtained and accurately characterized in terms of chemical properties. This knowledge allowed describing the mechanism of gold nanoparticles formation under the influence of plasma on the system.

## 2. Materials and Methods

### 2.1. Design of the Plasma Jets

**Figure 1:**
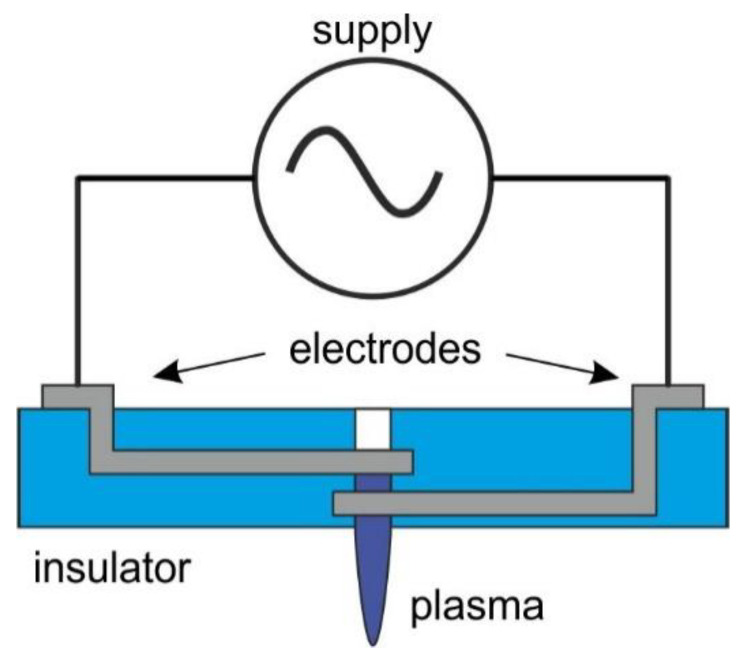
Construction of the plasma nozzle.

The structure of the nozzle is shown in Figure 1. The discharge was generated between the electrodes in a micro hollow cathode discharge (MHCD) configuration. The electrodes were located one above the other on subsequent layers. The discharge occurred in a cylindrical microcavity cut out in the center of the structure. To avoid etching and degradation of the metal electrodes, it was decided to protect them with a dielectric layer. Therefore, it was necessary to use an AC power supply.

**Figure 2:**
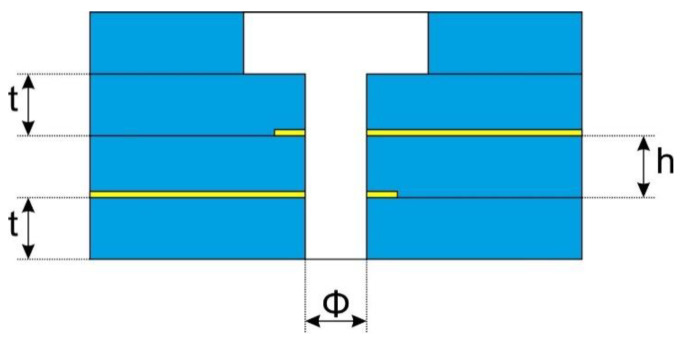
Geometric parameters of the nozzle.

The research involved determining how the nozzle structure affects the properties of the generated discharge. These parameters were: microhollow diameter Φ, distance between electrodes h and dielectric barrier thickness t. The cross section of the nozzle with the given dimensions is shown in Figure 2.

Ceramic structures were made of LTCC DuPont 951 (DuPont, Wilmington, NC, USA) ceramics and compatible pastes. The thickness of the ceramic foil used depended on the height of the recess and the thickness of the dielectric layers of the structure. These dimensions were treated as controllable factors in further experiments and are compiled in Table 1.

**Figure 3:**
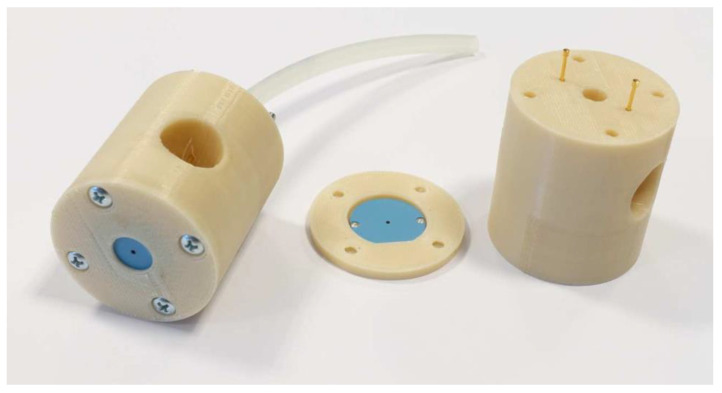
Fabricated plasma jets.

The pattern of each layer was laser-cut using LPKF Proto Laser U (LPKF, Garbsen, Germany). Metal layers were screen-printed using Aurel vs. 1520A (Aurel Automation SPA, Modigliana FC, Italy). All the layers were laminated for 20 min under 20 MPa pressure in the isostatic press and then green plasma jets were fired in a typical LTCC temperature profile for DuPont 951 system. Finally, the nozzles were placed inside polymer housing made in 3D printer. The plasma jet is presented in Figure 3.

### 2.2. Characterization of Plasma Properties

**Figure 4:**
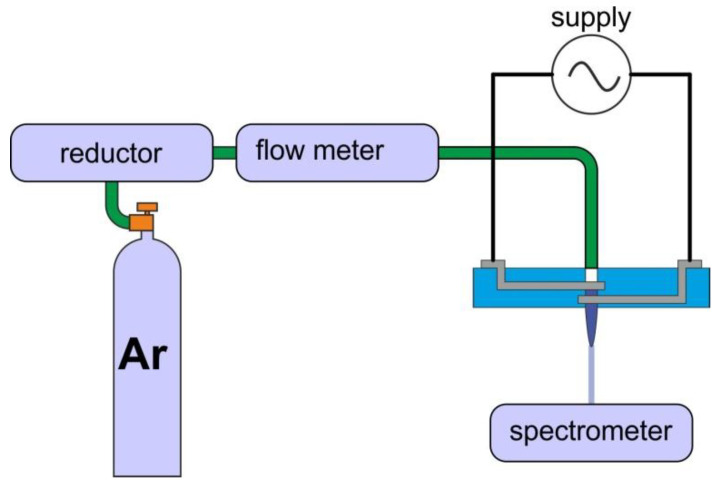
Measurement setup for determination of plasma properties.

The measurement system diagram is shown in Figure 4. The structures were connected to a high, alternating voltage source. It was possible to tune the following excitation parameters—peak-to-peak voltage U, frequency of the modulating signal f and duty cycle D, which determined the amount of power delivered to the plasma. In addition, it was possible to determine the impact of working gas flow rate using flow meter, as shown in Table 2. As mentioned earlier, the nozzles had geometries. It was also decided to study the impact of gas supply and gas flow, which gives 7 different parameters in total. The examination of each combination of these factors would result in 2187 experiments. Therefore, to reduce this number, methods of experiment design were used, and measurements were carried out according to Table 2. Combinations of factors were tested in random order to reduce the impact of systematic errors.

The basic method of plasma diagnostics is the analysis of emitted light using optical emission spectroscopy (OES). The spectra were recorded with a Shamrock SR500i spectrometer (Andor Technology Ltd, Belfast, Northern Ireland) with a Newton DU-920-OE CCD detector (Andor Technology Ltd, Belfast, Northern Ireland. The measurements were carried out using 1200 lines per mm and 1800 lines per mm diffraction gratings for the 200–400 and 400–800-nanometer range, respectively.

The most important parameters of cold atmospheric plasma include optical temperatures—T_rot_ rotational temperature and T_exc_ excitation temperature. They are used as approximations of the gas temperature for T_rot_ and T_exc_ electron energy.

The rotation temperature for nitrogen can be determined in several ways. The range from 354.5 to 357 nm was used here. If we plot the characteristic ln (I) = f (λ), then its slope will be proportional to T_rot_. For each experiment, such characteristics were plotted and after comparing them with the reference characteristics simulated in the LifBase 2.1 program [20], T_rot_ and T_exc_ were calculated. The intensities of the argon line were compared at 675.28 nm, 681.13 nm, 696.54 nm, 703.03 nm, 706.72 nm, 714.7 nm, 720.7 nm, 727.29 nm, 737.21 nm, 738.4 nm, 750.39 nm and 751.47 nm. These points were than used to calculate the slope, which is used to tabulate T_exc_.

### 2.3. AuNPs and PAW Synthesis

Gold nanoparticles (AuNPs) were obtained by chemical reduction of gold ions. Water solution of chloroauric acid ($HAuCl_{4}$) at a concentration of 0.08 mol/dm^3^ was used as a gold ions precursor. As a stabilizer, 1 mol/dm^3^ polyethyleneimine (PEI) (Sigma-Aldrich, Saint Louis, MO, USA) was used.

**Figure 5:**
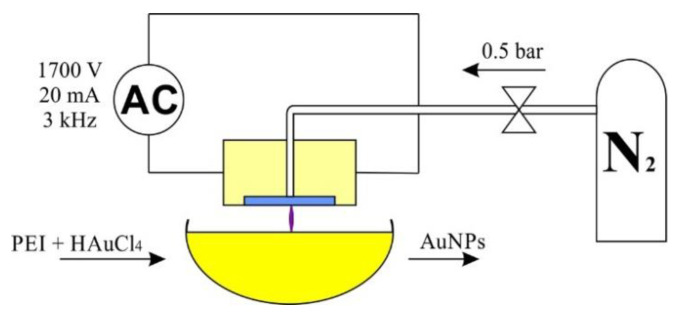
AuNPs reaction setup.

All syntheses were carried out at room temperature in DI water environment at a constant volume of 100 mL. To the water, 0.8 mL of PEI was added, and after 10 min of stirring 0.624 mL of $HAuCl_{4}$ was added. The concentration of gold nanoparticles was 100 ppm. The reaction setup is presented in Figure 5.

In case of the synthesis carried out without plasma, the sample was left without mixing and marked as reference. The samples were subjected to plasma treatment for 15 or 30 min at a reduced (0.5 bar) or increased (2 bar) plasma pressure and labeled: ref—reference; A—15 min, 0.5 bar; B—15 min 2 bar, C—30 min, 0.5 bar; D—30 min, 2 bar.

Plasma activated water was obtained under the same conditions and in the same system as the gold nanoparticles. Deionized water was subjected to plasma treatment. In case of PAW with the addition of PEI before plasma treatment, 0.8 mL of PEI was added to 100 mL of water.

Plasma activated water was investigated by the Shimadzu UV-1800 spectrophotometer (Kyoto, Japan). The absorption spectrum was measured in the range of 190 nm to 800 nm. The measurements were carried out in quartz cuvettes. The size distribution of the obtained gold nanoparticles was determined by dynamic light scattering (DLS) method. For this purpose, analyzer Zetasizer Nano ZS (Malvern Instruments, Malvern, UK) was used, which included a laser with a wavelength of 633 nm. To determine pH of colloids, Elmetron CPO-501 pH-meter (Elmetron, Zabrze, Poland) was used. The morphology of the nanoparticles was evaluated using a TEM microscope Fei Tecnai G2 X-Twin (Fei Company, Hillsboro, OR, USA).

## 3. Results

### 3.1. The Influence of the Geometry and Supply

**Figure 6:**
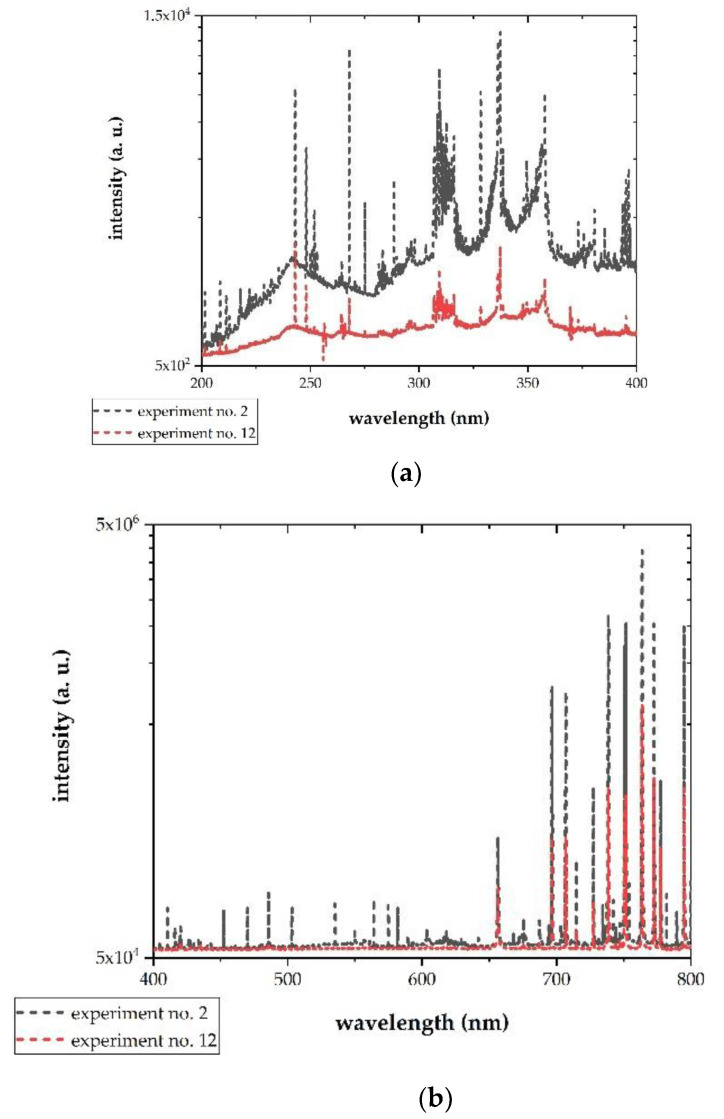
Spectra registered during experiments no. 2 and 12. (**a**) UV range and (**b**) VIS range.

The presence of plasma was observed for each combination of factors, which was determined by the appearance of argon lines and nitrogen bands. Recorded spectra differed in their shape, but in all cases it was possible to calculate temperatures as described in Section 2.2. Two examples are shown in Figure 6. The results of temperature calculations are presented in Table 3. As it can be seen, for higher T_exc_ the results are biased by a big error, yet they still provide qualitative information about plasma properties.

Using the L27 experiment table, it was possible to determine which factors have the strongest influence on the measured parameter. The results of such analysis are summarized in Table 4. The thickness of the dielectric layer had the biggest influence on the temperatures. The value of the excitation signal voltage strongly affected the excitation temperature (electron energy), with a small effect on the rotational temperature (temperature of the neutral gas background). In turn, by reducing the diameter of the aperture, the value of Trot was reduced. Proper selection of these two values can allow controlling the properties of microplasma generated in the nozzle. The interpretation is given in Section 4.

### 3.2. Production of PAW

As a result of water modification with plasma, plasma activated water (PAW) is formed with acidic pH and altered conductivity. These changes are caused by the presence of reactive oxygen (ROS) and nitrogen species (RNS). In case of the plasma obtained as a result of nitrogen ionization in the presence of oxygen, many primary particles are formed (atomic oxygen, singlet oxygen, peroxides, ozone, hydroxyl radicals, excited and atomic nitrogen), which then react to form secondary particles (including hydrogen peroxide, peroxynitrite, oxide nitrogen, nitrates and nitrite ions) [21,22]. As it has been reported the pH of PAW is drastically decreased with increasing the treatment time due to the formation of strong acids [23].

**Figure 7:**
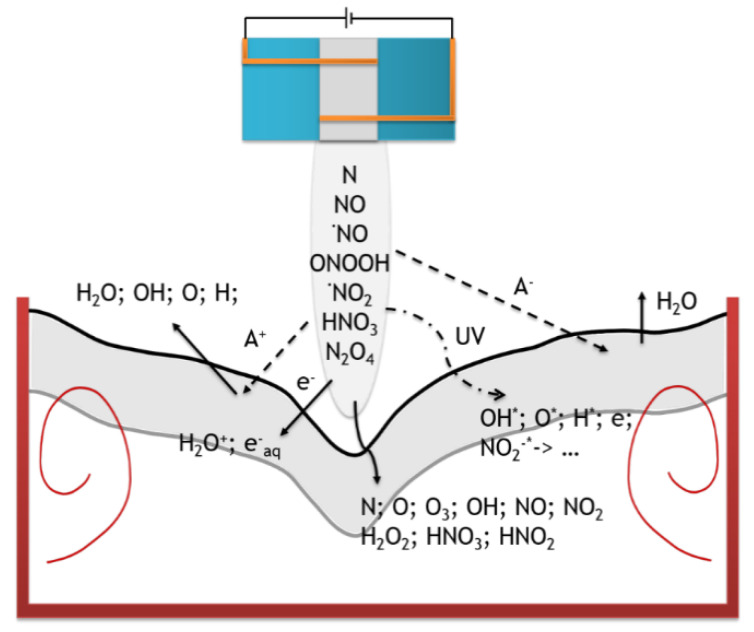
Phenomena occurring in a solution under the influence of plasma (gray marked—interface between gas and liquid).

During plasma treatment of an aqueous solution, many different reactions occur in it. Due to the plasma nozzle, the supplied gas (in this case nitrogen) undergoes ionization and it is the source of most particles supplied to the system. In the reaction system, the most important area is an interface between plasma and gas/liquid where most of the chemical reactions and physical phenomena take place. The reactive species are first produced in an atmospheric pressure plasma in the gas phase, these species are transported to the plasma–liquid interface, penetrate through and subsequently react in the liquid. The first phenomenon is the transportation of the mass of particles from a gas phase to a solution that occurs according to Henry’s law. It also depends on the plasma pressure, because according to Avogadro’s law the amount of ionizable particles also increases. In this case, ionization occurs at shorter distances, and local electromagnetic field disturbances may occur. In addition, photolysis occurs as a result of plasma exposure, which results in free radicals being formed in the solutions; cation emission leading to atomization of molecules from water, water evaporation, emission of anions and electrons resulting in the formation of hydrated electrons ($e_{aq}^{-})$ with very high reduction capacity (E° = −3 V vs. SHE) and a very short lifetime (Figure 7) [24,25].

There are two types of particles in plasma activated water: short-lived ($e^{-}$; $e_{aq}^{-}$; $e_{sd}^{-}$; atoms N and H; radicals $H^{\cdot }$), which disappear immediately after the plasma stops and long-lived (i.e., dissolved in water $H_{2}$; $N_{2}$ and $H_{2}O_{2}$), which may be present in the water for many weeks. Which of these particles can act as a reducer in the system will be determined by their potential (the lower, the better the reducer) and lifetime. Short-lived particles can only initiate a reaction, while long-lived particles can be responsible for the further progress of the reaction after the end of the plasma process.

**Figure 8:**
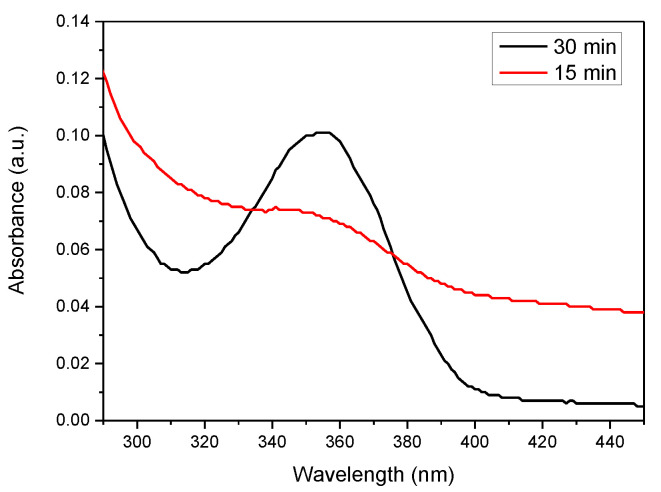
UV-Vis spectrum of water after plasma treatment.

APPJs was mounted above a glass baker with reaction mixture and worked under 2 bar $N_{2}$ pressure with 3 mm gap between the jet and liquid surface, which were tested experimentally. The main scope is the minimal gap, but the value is affected by the wetting angle of liquid. The influence of plasma on water is shown by UV-Vis spectroscopy (Figure 8) and its influence on water pH in Table 5. As a result of plasma treatment, the pH of the water decreased from 7 to about 3. A similar phenomenon is observed in case of an aqueous polyethyleneimine solution in which nanoparticles are obtained. Studies have shown that the pH of water containing PEI is about 10, and as a result of plasma exposure it significantly decreases to about 4.

Plasma water modification reduces its pH from neutral to acidic. This is due to the formation of nitric acid from $NO_{2}$ and NO formed in water as a result of plasma exposure. The presence of nitrogen groups and acids can be confirmed by UV-Vis measurements. Peaks in the 330–390 range are characteristic for $HNO_{2}$ and the peak at 302 nm for $HNO_{3}$ [25]. In most cases in PAW, both acids are present, and their ratio depends on the power, duration of action and type of plasma. According to Kojtari at al. [26], the concentration of $HNO_{3}$ increased and the concentration of $HNO_{2}$ decreased with the increase in the duration of plasma modification. These results suggested that the plasma treated water had significantly strong oxidative characteristics.

Analyzing the results of UV-Vis (Figure 7) the concentration of acids in water can be calculated with the Lambert–Beer’s law (1):

(1) A=εbc

where: *A*—measured absorbance, *b*—light path length (1 cm), *c*—concentration of substance, ε—the molar absorptivity or extinction coefficient: ε ($HNO_{3}$) = 70 M^−1^ cm^−1^ in 300 nm and ε $HNO_{2}$) = 23 M^−1^ cm^−1^ in 354 nm [27]. Our research indicates that water after 30 min of plasma action contains 4.39 mM nitrous acid—$HNO_{2}$ and trace amounts of nitric acid—$HNO_{3}$. Nitrous acid is a weak acid, which means that it does not dissociate completely. Its dissociation constant (*K*) is 4.5 × 10^−4^. The pH of the plasma treated deionized water is 3.06 and is described by the Equation (2):

(2) $$pH=-\log\left[H^{+}\right]$$

where: $\left[H^{+}\right]-$ concentration $H^{+}$ (mol/dm^3^). It follows that the concentration of $H^{+}$ and $NO_{2}^{-}$ is about 1×10^−3^ mol/dm^3^. The dissociation constant is the ratio of the product of dissociated ions to undissociated molecules raised to respective powers and for nitrous acid it is described by the Equation (3):

(3) $$K=\frac{\left[H^{+}\right]\left[NO_{2}^{-}\right]}{c-\left[H^{+}\right]}≅\;3\times 10^{-4}$$

In Equation (3), after substitution with nitrous acid concentration determined on the basis of Lambert–Beer law and dissociated ion concentration determined on the basis of pH measurements, a dissociation constant value of about 3 × 10^−4^ was obtained, which is very similar to the literature data [28,29].

### 3.3. AuNPs Syntheses

**Figure 9:**
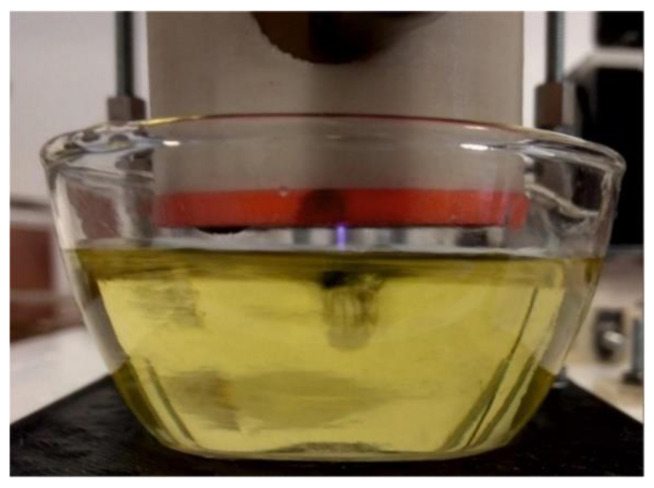
Plasma jets (PJs) under nitrogen working above the surface of liquidous solution.

AuNPs were obtained from reaction of gold ions $Au^{3+}$. The precursor of the reaction was 0.08 M chloroauric acid ($HAuCl_{4}$). The plasma jet under nitrogen operation in polymer chassis is presented in Figure 9. After the synthesis, all colloids were subjected to a pH test (Table 6), observations were made using TEM and DLS tests. The progress of the reaction was controlled by UV-Vis absorption studies.

### 3.4. Characterization of AuNPs

**Figure 10:**
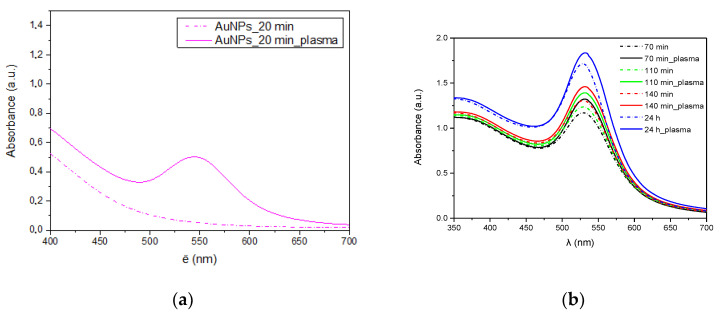
UV-Vis spectra of AuNPs. (**a**) after 20 min; (**b**) all synthesis time.

During the synthesis of gold nanoparticles, UV-Vis analyses of the obtained colloids were performed at specific intervals. A characteristic peak of gold nanoparticles around 520 nm was observed in the spectra of colloids obtained in the presence of plasma and a reference sample (Figure 10). In case of the synthesis carried out in the presence of plasma, the reaction began much faster (Figure 10a) as evidenced by the higher peak characteristic of gold plasmon. A higher concentration of gold nanoparticles in the solution is visible throughout the synthesis (Figure 10b). However, this difference is getting smaller as the reaction progresses.

**Figure 11:**
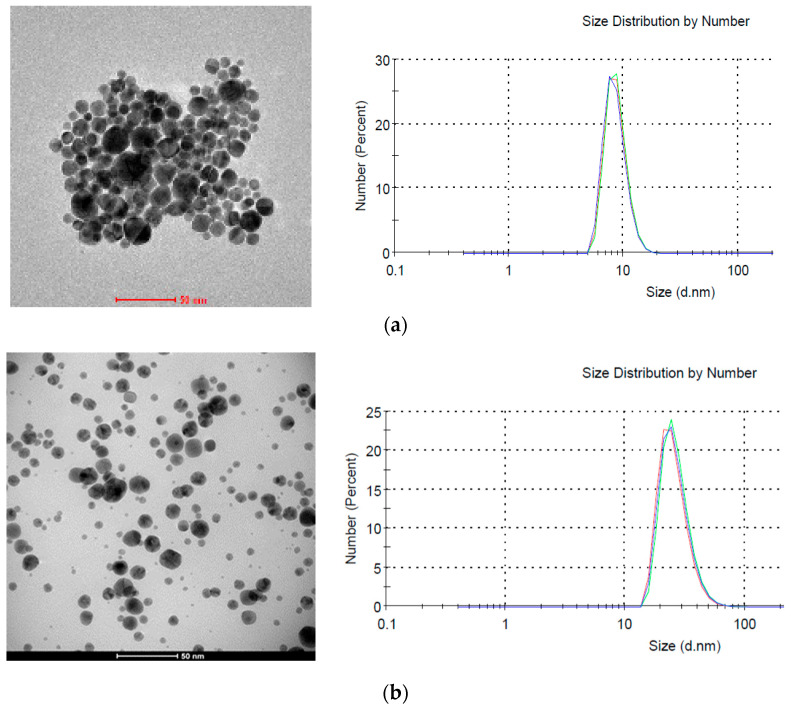
HRTEM images and dynamic light scattering (DLS) images of AuNPs. (**a**) Reference; (**b**) synthesized in plasma (15 min, 0.5 bar).

According to DLS research the average size of nanoparticles synthesized without plasma was about 8 nm (Figure 11a), while the size of those synthesized in the presence of plasma was about 25 nm (Figure 11b). However, the difference in size calculated from HRTEM images is smaller and for both samples it is about 18 nm. This difference results from a different way of measuring samples. In HRTEM nanoparticles are examined after drying, while in DLS directly in the colloid. Additionally, the hydrodynamic diameter is tested in DLS, which can also take into account the size of the polymer micelles located around gold nanoparticles. This is also in line with the UV-Vis tests (Figure 10) where no absorption peak shifts were observed depending on the plasma application.

**Figure 12:**
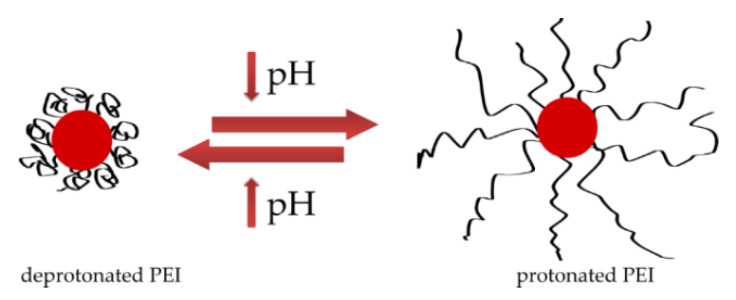
Shape of polyethyleneimine at different pH levels.

A different stabilizing polymer structure may be responsible for the difference in nanoparticle size observed in DLS results. Both colloids are stabilized with polyethyleneimine, which in the reference sample with a pH of 10 is in a deprotonated form, its chain is coiled and it stabilizes nanoparticles, while the pH of the colloid obtained in the plasma is much lower, which leads to protonation of PEI and straightening of the polymer chain (Figure 12) [30,31]. The change in the structure of the PEI chain is directly related to its ability to stabilize particles. However, as mentioned earlier, nanoparticles with a negative surface charge are formed because of the plasma [15]. Negatively charged surface of nanoparticles interacts with positive charges of protonated polyethyleneimine chains, resulting in the formation of a connection between nanoparticle and polymer and the particles are effectively stabilized.

**Figure 13:**
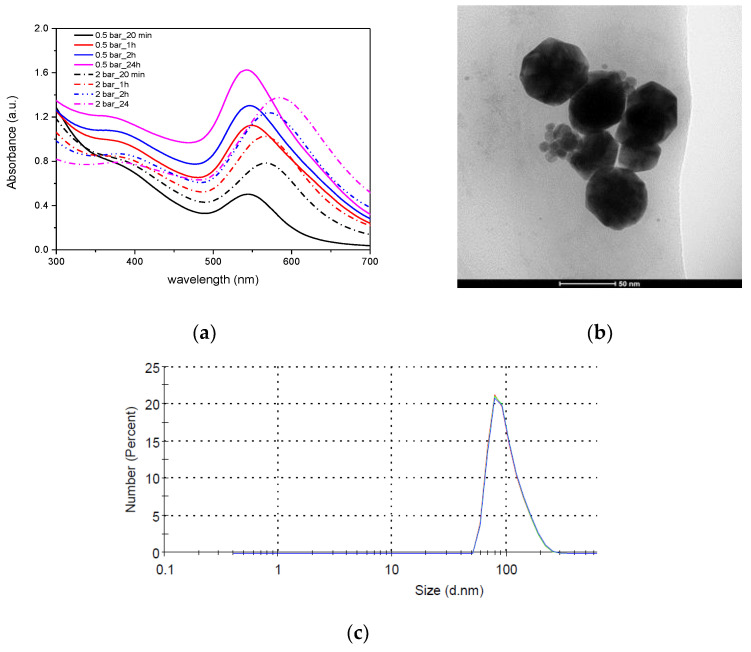
AuNPs synthesized in high-pressure plasma. (**a**) UV-Vis, (**b**) shape presented in TEM and (**c**) DLS image of nanoparticles.

The design of the developed plasma generators makes it possible to obtain microplasma not only at an atmospheric pressure, but also at an elevated pressure. For this reason, studies on the effect of plasma pressure on gold nanoparticle formation were performed. At a higher plasma pressure (2 bar), the synthesis begins faster, but the absorption peak of the resulting colloid after the synthesis is lower and wider (FWHM = 119) and clearly shifted towards larger wavelengths (588 nm) (Figure 13a). The particles obtained at a lower plasma pressure (0.5 bar) had a peak at a wavelength of 546 nm (FWHM = 100 nm). The change in the size and shape of nanoparticles is confirmed by TEM images (Figure 13b) and DLS (Figure 13c, Table 4), which show that the diameter of nanoparticles is about 50 nm, their shape is hexagonal with clearly shaped side walls. Planes (111) and (100) are visible in TEM images. This shape of gold nanoparticles is characteristic of metallic particles with *fcc* structure. Usually, AuNPs with hexagonal (icosahedral) profiles are formed in the presence of cetyltrimethylammonium bromide (CTAB), which is a surfactant used in preparing non-spherical AuNPs. The anisotropic shape of nanoparticles is a result of the preferential binding of the cationic surfactant (CTAB) functional group to the surface (100) of AuNP nucleus [32].

The use of a plasma reactor and synthesis with appropriate parameters also allows obtaining nanoparticles with non-spherical shapes without the need for surfactants. The resulting particles can be used, among others, in catalysis, because they increase the efficiency of the catalyzed process [33,34].

## 4. Discussion

As it can be seen in Table 7, the optical temperatures have changed significantly in individual experiments. The lowest measured T_rot_ was 506 K, the highest 739 K with a median of 633 K and the mean value close to it 623 K. For T_exc_, the lowest recorded value is 4186 K, the median is 4986 K, average value 6472 K, and the highest value is 18,509 K. However, it should be noted that the latter value is subject to a very high measurement uncertainty. This is due to the fact that for such a high temperature, the T_exc_ model loses its accuracy. Nevertheless, in some experiments this temperature exceeded 10,000 K, despite these limitations.

The use of the L27 table allows the estimation of the influence of individual factors on the measured values. This is done by averaging the measured values for those measurements where the factor took a certain level (low, medium or high). For example, for parameter t, average values are calculated from experiments 1–9 for low level, 10–18 for medium level and 19–27 for high level (see Table 4). The calculated values are summarized in Table 6. The phenomena occurring in the microplasma are complex and related to each other. However, the obtained results allow making some interpretations.

With increasing thickness of the dielectric barrier t, discharge current and electric field density decrease. As a result, the power delivered to the plasma is limited and so is T_rot_. On the other hand, T_exc_ increases, because with a lower electron density and shorter mean free path, collisions between electrons and other particles are less frequent. Therefore, energy transfer from electrons decreases, which elevates T_exc_. For the rest of geometric parameters, namely height h and diameter Φ relations are not so simple. Basically, smaller dimensions increase power density and T_rot_. Interestingly, the lowest T_exc_ is obtained for medium factor levels. This is probably because, for smallest dimension, high electric field density accelerates electrons to higher energies. Above a certain point, despite lower field density, a low collision rate allows electrons to gather and maintain energy.

By increasing the voltage U, the power increases, and hence the gas temperature, i.e., T_rot_. Interestingly, T_exc_ for the highest level decreases, probably because electrons with too much energy are thrown out of the inter-electrode area, which causes a negative energy balance. The frequency of modulation **f** mainly affects the propagation of acoustic waves in the discharge that locally change the density of the gas. At the lowest frequency, the areas of reduced pressure are the largest, which is why electrons without collisions can increase their T_exc_. After obtaining it, they fall into an area of higher pressure, where the energy of electrons increases the energy of gas (T_rot_). This transfer is increased until the ions stop following the increasing frequency and reduce the T_rot_ and the electrons accumulate T_exc_ in less frequent collisions.

Increasing the duty cycle D boosts the power delivered to the plasma. It results in a T_rot_ increase. For D at a medium level (50% fill), a minimum of T_exc_ is observed. Most likely, such a low-frequency component throws some of the electrons out of the discharge area, while 30% and 70% values have more high-frequency signal components.

Finally, increasing the flow rate **v** increases the pressure and thus gas density, which causes its temperature to grow due to more frequent collisions. On the other hand, above a certain threshold, a decrease in T_rot_ is observed, because the elevating pressure reduces the efficiency of ionization by electrons, which is perfectly visible in a lower T_exc_. Of course, as the **v** increases, so does the number of electrons removed from the micro cavity.

The study presents a method that enables obtaining of gold nanoparticles under the influence of nitrogen plasma. Optimization of such a process and its control requires thorough knowledge of the mechanism of nanoparticle formation in the solution. First of all, it is necessary to determine what particles play the role of a reducing agent in such a system.

The standard electrochemical potential of $Au^{3+}$ reduction to metallic $Au^{0}$ is 1.498 V (4) [35]. In case of the synthesis carried out without the presence of plasma, the role of a reducer in the system can be performed by $OH^{-}$ ions whose standard electrochemical potential is 0.401 V (5). This is a very likely reaction because the basicity of the solution containing polyethyleneimine is about 10. Thus, the reduction reaction may occur as a result (6).

(4) $$E^{o}=1.498\;V\;Au^{3+}+3e^{-}=Au$$

(5) $$E^{o}=0.401\;V\;O_{2}+2H_{2}O+4e^{-}=4OH^{-}$$

(6) $$4AuCl_{4}^{-}+12OH^{-}=4Au+16Cl^{-}+6H_{2}O+3O_{2}$$

However, in case of the plasma synthesis, the reaction occurs in an acidic environment in which $OH^{-}$ ions cannot act as a reducing agent because they are consumed in the neutralization reaction. As a result of plasma exposure, hydrated electrons, radicals, ions and various nitrogen compounds, which can act as a gold-ion reducer, are present in the solution. These particles, as it has previously been mentioned, fall into two categories: short-term—only present during plasma operation and long-term—present in the solution much longer after the end of the plasma processing. Due to this fact, the reduction of gold ions does not occur rapidly in the solution and, as it occurs long after the end of plasma plating, it is most likely that the reduction of gold ions is mainly caused by the long-lived particles, and it is among them that the reducing agent should be sought.

In addition, Chen et al. [36] showed that the reduction of gold ions in plasma is mainly due to the long-lived particles, and short-lived particles have a much smaller impact. The research was carried out on the basis of a comparison of the synthesis of gold nanoparticles from a chloroauric acid solution directly during plasma operation and in plasma activated water (PAW). The reaction carried out in the plasma was slightly faster than that taking place in the PAW.

In order to determine which of the long-lived particles can act as a reducing agent in the system, it is necessary to take into account the properties (oxidizing/reducing) of individual particles and their standard electrochemical potential $E^{o}$ because, only a particle whose potential will be lower than the potential for gold reduction can be an effective reducer. For this reason, the $H_{2}O_{2}$ hydrogen peroxide present in the solution cannot act as a gold-ion reducer, because it is a very strong oxidant, especially in an acidic environment ($E^{o}=1.77\;V$). It can act as a reducing agent only in the presence of very strong oxidants, such as potassium permanganate [29].

Nitrogen oxide NO, present in the solution, is the compound with a sufficiently low potential ($E^{o}=0.983\;V$) (7). In addition, the electron located on the π* orbital in the NO molecule easily breaks off to form a nitrosyl ion $NO^{+}$ [29]. A relatively easy release of the electron indicates that this compound is a good reducer and can act as one in the examined system.

(7) $$E{}^{\circ}=0.983\;V\;HNO_{2}+H^{+}+e^{-}=NO+H_{2}O$$

Nitrous acid is formed as a result of plasma exposure to water, which was confirmed by UV-Vis tests (Figure 8) and it is also the final equation (8) product. Depending on the conditions, it can have both reducing and oxidizing properties. However, in an acid solution in the presence of oxidants (in this case gold ions and hydrogen peroxide) it can be oxidized to nitric acid according to the reaction (5).

(8) $$E{}^{\circ}=0.934\;V\;NO_{3}^{-}+3H^{+}+2e^{-}=HNO_{2}+H_{2}O$$

Considering the reaction potential ($E^{o}=0.934\;V$), nitrous acid can also act as a reducing agent in the tested system. Thus, the reduction of gold ions in water, under the influence of cold nitrogen plasma, can occur according to the Equations (9) and (10):

(9) $$Au^{3+}+3NO_{2}+3H_{2}O=Au+3HNO_{3}+3H^{+}$$

(10) $$2Au^{3+}+3HNO_{2}+3H_{2}O=2Au+3NO_{3}^{-}+9H^{+}$$

In addition, it is well known that Gibbs energy $G^{o}$ will determine the direction of a reaction. When $G^{o}$< 0, the reaction occurs spontaneously. It depends on the standard electrochemical potential and is described by the Equation (11):

(11) $$\Delta G^{o}=-nF\Delta E^{o}$$

where: *n*—the number of electrons exchanged in the reaction, F—Faraday constant (96,486.7 C/mol).

Gibbs energy of both reactions (9) and (10) is negative, which means that these reactions occur spontaneously. In the reaction in which the reducing agent is nitric oxide (9) $G^{o}$ = −149 kcal/mol and nitrous acid (10) $G^{o}$ is −163 kcal/mol.

## 5. Conclusions

Two possible applications of plasma jets were presented in this article: AuNPS production without heavy species and production of the PAW. Optical methods were used to describe the species present in plasma and the results obtained in nanoparticles synthesis. The spectra emitted during the discharges showed that the OH groups were present during all measurements, which confirms the theory for PAW [22]. The results obtained for PAW were very similar to those in a different work utilizing plasma generated in LTCC ceramic construction [7]. The main differences were the non-flowing system of PAW production. The main reactions were based on hydrogen, oxygen, nitrogen and free electrons which were accelerated in plasma. The optimization of APPJs was developed using the Tauguchi experiment method (L27). The results show that the thickness of the dielectric barrier had the biggest influence on the optical temperatures. Overheating of APPJs was not observed.

The plasma jets made in LTCC technology enable obtaining gold nanoparticles without the need for additional reducing agents and in a shorter time than in the process without the used of plasma. Depending on the conditions of the process, gold nanoparticles of spherical shape or with hexagonal (icosahedral) profiles were formed. In the tested system, the ionized gas was nitrogen. For this reason, many reactive forms of nitrogen were present in the system, and after adding plasma the pH of the solution was low (around 3) and the presence of high concentration nitrous acid was detected.

The reduction mechanism of gold ions largely depended on the pH of the solution, which strongly decreased under the influence of plasma. The change in pH also affected the structure of the stabilizing polymer. In low pH solutions, polyethyleneimine protonation occurred, resulting in chain straightening. As a consequence, the mechanism of stabilizing nanoparticles with PEI changed. Electrostatic interactions were formed between protonated PEI chains and negatively charged gold nanoparticles.

An important scientific goal of the publication was to determine which compound formed from plasma has the role of a gold-ion reducer. Based on the literature analysis, UV-Vis measurements, analysis of thermodynamic data and redox potentials, it was found that the reduction of gold ions is caused by the presence of nitric oxide NO and nitrous acid $HNO_{2}$. This is evidenced by the values of standard electrochemical potentials, Gibbs free energy and the presence of an electron located on the π* orbital in the NO molecule, which easily breaks off.

## Figures and Tables

**Table 1:** Values of geometric parameters.

Level	t (µm)	h (µm)	Φ (µm)
(low) 1	100	100	300
(normal) 2	150	150	600
(high) 3	210	210	900

**Table 2:** Factor levels of the experiment parameters for Taguchi analysis.

Level	U (kV)	f (kHz)	D (%)	v (sccm)
(low) 1	6.5	1	30	30
(normal) 2	7	1.5	50	60
(high) 3	7.5	2	70	90

**Table 3:** Factor levels of the experiment parameters for Taguchi analysis.

Experiment	T_rot_ (K)	ΔT_rot_ (K)	T_exc_ (K)	ΔT_exc_ (K)
1	647	39	4555	162
2	666	36	4809	242
3	633	36	4635	127
4	506	16	13,779	2380
5	676	35	5088	215
6	671	35	4864	91
7	629	32	7013	1879
8	626	31	4986	156
9	577	30	5034	178
10	726	53	4729	131
11	667	37	4729	149
12	739	42	4659	163
13	635	35	4819	151
14	700	43	4757	199
15	508	21	4955	305
16	683	36	4991	141
17	680	33	4186	747
18	679	35	4551	161
19	631	35	16,247	2757
20	552	25	9959	778
21	545	24	6430	2367
22	638	37	5585	323
23	609	24	5391	403
24	542	16	18,509	17,511
25	538	20	5078	207
26	582	24	4984	240
27	525	21	5426	456

**Table 4:** Influence of parameters on discharge (1—biggest impact, 7—lowest impact).

Temperature	t	h	Φ	U	f	D	v
T_rot_	1	4	2	5	3	6	7
T_exc_	1	3	6	2	5	7	4

**Table 5:** pH of plasma treated water.

Time of Plasma Treatment (min)	Water	Water + PEI
0	7.00	10.12
15	3.60	7.15
30	3.06	4.12

**Table 6:** pH after plasma treatment.

Parameters	Reference	Experiment			
		A	B	C	D
Time of plasma treatment (min)	0	15	30	15	30
pH	3.6	3.6	3.6	3.5	3.4
Size (nm)	11 ± 3	25 ± 7	33 ± 9	100 ± 35	70 ± 15
Pressure (bar)	-	0.5	0.5	2.0	2.0

**Table 7:** Calculated mean values of T_rot_ and T_exc_ for each level of investigated factors.

Temperature	Level	t	h	Φ	U	f	D	v
T_rot_ [K]	1	625.6	641.9	641.9	603.7	626.0	605.7	604.4
	2	668.5	593.4	625.5	624.8	639.5	623.8	633.5
	3	573.4	606.9	600.1	639.1	602.0	637.1	629.6
T_exc_ [K]	1	6084.7	6356.8	6356.8	4890.9	7421.7	6649.5	7709.2
	2	4708.4	5972.2	5926.3	7788.4	5432.2	5126.0	6217.6
	3	8623.3	6783.1	7133.3	6737.1	6562.5	6123.8	5489.6
